# Dual SOT Switching Modes in a Single Device Geometry for Neuromorphic Computing

**DOI:** 10.1021/acs.nanolett.5c01100

**Published:** 2025-04-17

**Authors:** Abhijeet Ranjan, Tamkeen Farooq, Chong-Chi Chi, Hsin-Ya Sung, Rudis Ismael Salinas Padilla, Po-Hung Lin, Wen-Wei Wu, Ming-Yen Lu, Rahul Mishra, Chih-Huang Lai

**Affiliations:** † Department of Materials Science and Engineering, National Tsing Hua University, Hsinchu 30013, Taiwan; ‡ Centre for Applied Research in Electronics, Indian Institute of Technology Delhi, New Delhi 110016, India; § Instrumentation Center, National Tsing Hua University, Hsinchu 300, Taiwan; ∥ College of Semiconductor Research, National Tsing Hua University, Hsinchu 30013, Taiwan; ⊥ Department of Materials Science and Engineering, National Yang Ming Chiao Tung University, Hsinchu 300, Taiwan; # Future Semiconductor Technology Research Center, Hsinchu 30078, Taiwan

**Keywords:** Spin−Orbit Torque, Binary Switching, Multilevel Switching, Neuromorphic Computing, Artificial Neurons, Artificial Synapses

## Abstract

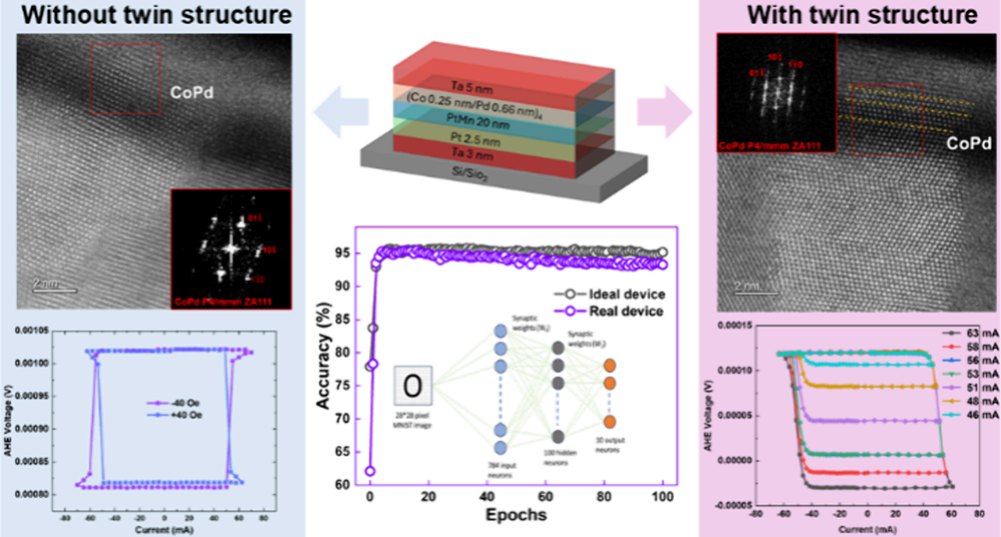

Neuromorphic computing aims to replicate the brain’s efficient processing through artificial neurons and synapses, requiring binary and multilevel switching. We present a PtMn/(Co/Pd)_4_/Ta device that uniquely enables dual spin–orbit torque (SOT) switching modes—binary and multilevel (analog)—within the same geometry and stack structure, eliminating the need for device modifications. Binary SOT switching is achieved via domain wall nucleation and propagation at moderate current levels (∼65 mA), while multilevel switching occurs via domain nucleation mode without significant propagation after a high-current treatment (∼85 mA). The transition between two modes originates from structural changes after the current treatment. These modes allow for neuronal and synaptic functionalities, with the device achieving 96% accuracy in digit/letter recognition on the MNIST data set using an artificial neural network (ANN). The device’s robust perpendicular magnetic anisotropy (PMA), dual-mode switching under a small in-plane field (*H*_X_), and simplified fabrication underscore its promise as an energy-efficient solution for neuromorphic computing.

Spin–orbit torque magneto resistive random access memory (SOT-MRAM) represents a promising nonvolatile memory technology utilizing heavy metal/ferromagnet (HM/FM) heterostructures.^1−9^ Heavy metals with strong spin–orbit coupling facilitate the magnetization switching of the ferromagnetic layer through mechanisms such as the bulk spin-Hall effect (SHE) and interfacial Rashba–Edelstein effects.^1−9^ The potential of SOT-based spintronics devices extends beyond memory applications, drawing attention to their applications in energy-efficient brain-inspired neuromorphic computing and memristive systems.^10−30^ Neuromorphic computing aims to emulate the brain’s architecture and functionalities by employing artificial neurons and synapses for complex cognitive tasks. In this framework, artificial neurons process incoming signals and activate upon reaching a specific threshold, while artificial synapses modulate their strength to influence signal transmission, thereby mirroring the plasticity inherent in biological systems.^10−29^ This bioinspired paradigm seeks to address the limitations posed by conventional computing architectures, particularly the von Neumann bottleneck, which separates memory from processing and contributes to inefficiencies in speed and energy consumption.^10−29^

SOT switching can be categorized into binary and multilevel modes,^1−11^ with some systems exhibiting stochastic switching.^22,23^ Binary SOT switching is prevalent in memory applications. In addition, Kurenkov et al. have utilized it as a neuronal element in neuromorphic computing.^10^ Stochastic switching has been found useful in applications such as physically unclonable functions (PUFs)^22^ and neuromorphic computing,^23^ while analog switching serves primarily as the synaptic element.^10−29^ These diverse switching modes can be achieved by tuning stack structures and device geometries, allowing for the manipulation of magnetic properties and parameters such as exchange bias and Dzyaloshinskii–Moriya interaction (DMI), ultimately facilitating the modulation of SOT switching mechanisms.^10,11,22,25,27,28^

Recent advancements in SOT research have utilized antiferromagnet/ferromagnet (AFM/FM) heterostructures to demonstrate field-free SOT switching,^10,11^ electrical switching of exchange bias (EB) using SOT,^30^ utilization of the AFM/FM interface for neuromorphic and stochastic applications,^10,11,22,30−33^ etc. Notably, Pt_1–*x*_Mn_*x*_ has demonstrated thermal robustness that withstands back-end-of-line (BEOL) processing temperatures and facilitates the SOT efficiency comparable to that of Pt and much lower DMI than Pt.^33−36^

This work presents a (Co/Pd)_4_ multilayer on Pt_50_Mn_50_ with strong perpendicular magnetic anisotropy, achieving dual SOT switching modes via current modulation. Kurenkov et al. explored PtMn/(Co/Ni)_*n*_ heterostructures to demonstrate artificial neurons and synapses with varying device sizes to achieve these dual modes.^10,11^ Cao et al. transformed a binary perpendicular ferromagnet (Pt/Co/Ta) into a multilevel device by introducing an additional in-plane Co layer (Pt/Co(OP)/Ta/Co(IP)), enabling synaptic plasticity through spin–orbit torque-driven domain wall motion.^28^ However, these approaches necessitate geometrical and stack structure modifications, respectively, to switch between binary and multilevel behaviors.^10,28^ To our knowledge, our work is the first demonstration of dual-mode functionality in fully fabricated SOT devices without device geometry and stack structure modifications. Initially, the device operates in binary mode for memory and neuron-like functions. After a high current treatment, it transitions to a multilevel mode, functioning as a synaptic element for neuromorphic computing. We demonstrated that the transition originates from the structural changes after current treatment. Integrated into a three-layer artificial neural network (ANN), it achieves 96% accuracy on MNIST letter/digit recognition. This dual-mode architecture simplifies fabrication, reducing costs and enabling versatile spintronic applications.

Figure 1(a) illustrates the heterostructure employed in this study. Magnetic properties of the heterostructure are shown in Supporting Information S1. The loops exhibit robust perpendicular magnetic anisotropy with an anisotropy field (*H*_K_) exceeding 1 T, which remains consistent before and after annealing. Because we annealed the sample without a magnetic field, we did not observe exchange bias but rather enhanced coercivity. Figure 1(b) displays an optical micrograph of the Hall cross devices, accompanied by the setup for typical SOT tests utilizing the anomalous Hall effect (AHE) as the readout signal. The micrometer-sized Hall cross retains robust perpendicular magnetic anisotropy (PMA), as evidenced by the squareness of the field-swept AHE (Figure 1(c)). Subsequently, we conducted SOT switching tests, as presented in Figure 1(d). We applied 300 μs pulses with increasing current amplitudes, reading the magnetization state after each pulse. A current of ±65 mA facilitated reversible magnetization switching from up to down or down to up, contingent upon the direction of the applied field along the current direction (*H*_X_). A modest *H*_X_ of 40 Oe sufficed for complete SOT switching with the Pt-like polarity. Note that the thin Pd generates a negligible spin current.^37,38^ The SOT switching polarity confirms the SHE originated from bottom PtMn^33−36^ and top Ta.^7^ The rapid and sharp switching observed aligns with the performance expected from HM/FM heterostructures, distinguishing it from the PtMn/(Co/Ni)_*n*_ systems reported by Fukami et al., where magnetization switched in a more gradual manner due to the influence of in-plane exchange bias.^10,11^ The reduced *H*_X_ required for switching corroborates that the DMI at the PtMn/Co interface is significantly smaller than that in Pt/Co.^36^ We performed *H*_X_ dependence of SOT to quantitatively estimate the DMI field for PtMn and Pt cases. The detailed discussion is given in Supporting Information S2, which indeed verifies DMI in PtMn/Co is much smaller than that at the Pt/Co interface. Recognizing heat generated during the SOT process is critical for magnetization switching dynamics, we further investigated the impact of increasing input current amplitude while maintaining a constant pulse width (300 μs) on the switching behavior of our devices. As illustrated in Figure 1(e), upon reaching an input current amplitude of 85 mA, we observed a substantially enhanced resistance, indicated by the measured resistance in the current channel resistance (*R*_xx_). Following this high-current treatment, the magnetization exhibited a gradual switching behavior akin to that reported by Fukami et al. in their micron-sized devices featuring PtMn/(Co/Ni) multilayers.^10,11,25^ The multilevel (analog) SOT switching behavior, under *H*_X_ = 40 Oe, is depicted in Figure 1(f). This enhancement of device resistance and transition from binary SOT switching to multilevel SOT after a high current treatment are irreversible. The transition mechanism will be discussed later. The stability and reproducibility of the intermediate magnetization states of the multilevel are shown in Supporting Information S3.

**Figure 1:**
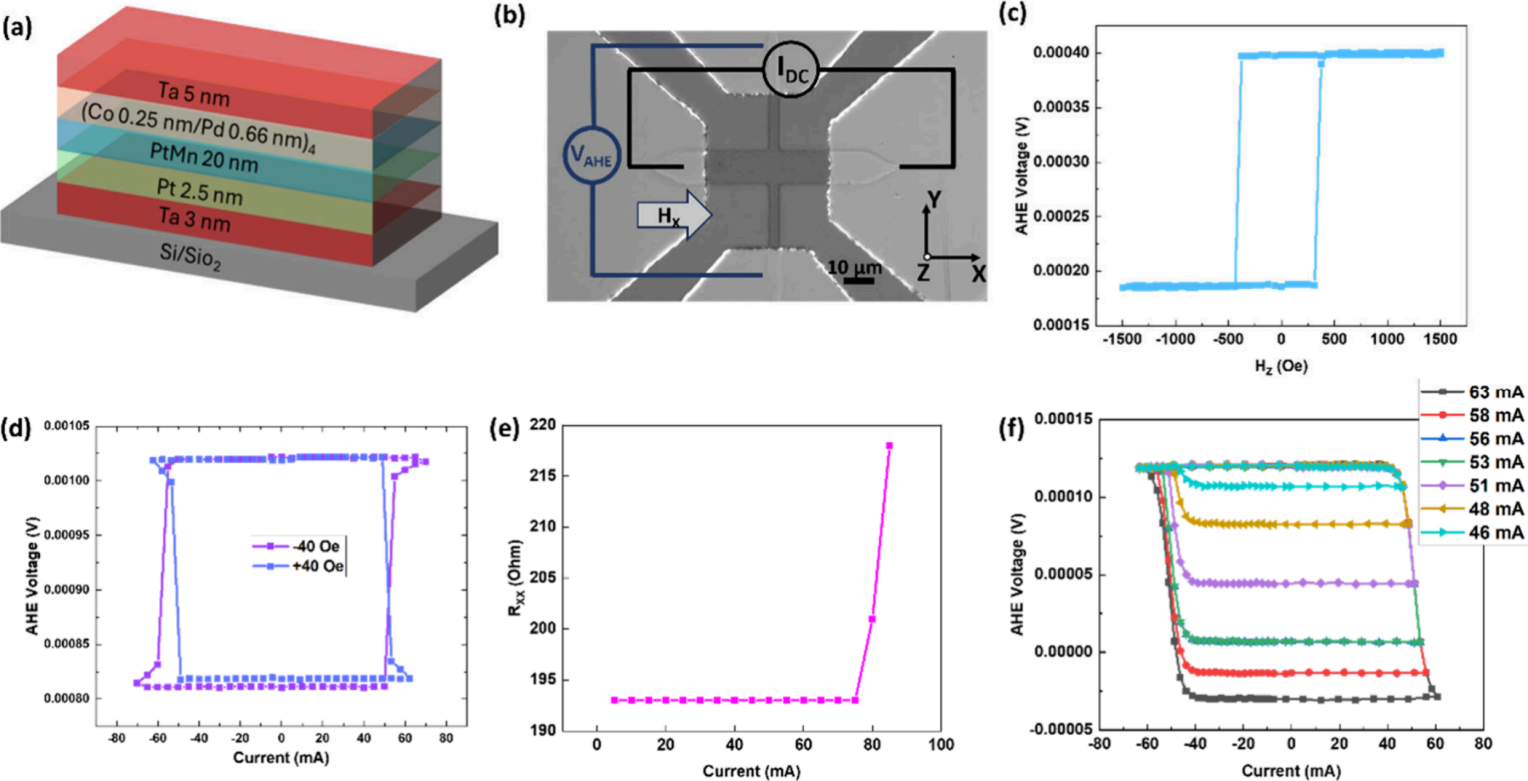
SOT switching in PtMn/(Co/Pd)_4_. (a) The layer structure used in this study. (b) Optical micrograph of the fabricated device and SOT switching setup. (c) AHE loop by field switching. (d) Current-induced binary SOT (±65 mA) switching under *H*_X_ = ±40 Oe. (e) Variations of resistance with the input current along the current channel (*R*_XX_). (f) Analog switching with *H*_X_ = 40 Oe after large current treatment (85 mA). The pulse width is 300 μs for each pulse during SOT switching.

Thus, we demonstrate that our devices achieve dual SOT switching modes, binary and multilevel, in the same device, contingent upon prior exposure to large current pretreatment. Remarkably, both as-deposited and annealed samples exhibited identical dual switching modes before and after the current treatment. This finding contrasts sharply with the observations reported by Kurenkov et al. in which distinct switching modes in PtMn/(Co/Ni)_*n*_ heterostructures required certain PtMn thickness and different device sizes for dual modes.^10,11,25^ The different modes were attributed to PtMn anisotropy and pinning strength on Co/Ni domains by PtMn. Later, we will show that the dual modes in our devices can be observed in different PtMn thicknesses or even without PtMn; that is, unlike previous reported works, the coupling between PtMn and the Co/Pd multilayer does not play the key role for our dual modes.

To confirm stable binary and analog switching, we ensured binary SOT switching remained consistent under varying conditions, including pulse width, external field (*H*_*x*_), and repeated cycles. Maintaining a clear threshold between the critical current for binary switching (∼65 mA) and the binary-to-analog transition (∼85 mA) prevents inadvertent transitions. This distinct current difference highlights the robustness of binary switching against input variations.

We tested binary switching stability by varying the pulse width from 300 μs to 100 ms at 65 mA (Figure 2(a)). The switching remained binary throughout. As shown in Supporting Information S4, we also performed the test for shorter pulse widths (10 to 300 μs) and the switching remained binary as well. Similarly, binary behavior persisted across an *H*_*x*_ range from tens of Oe to 600 Oe (Figure 2(b)). Reproducibility was confirmed through 1000 binary SOT switching cycles at ±65 mA (Figure 2(c)), after which the SOT curve still showed binary switching (Figure 2(d)). These results demonstrate the robustness of binary SOT switching under varied conditions, transitioning to multilevel only at significantly higher currents (∼85 mA), ensuring reliable operation for practical applications.

**Figure 2:**
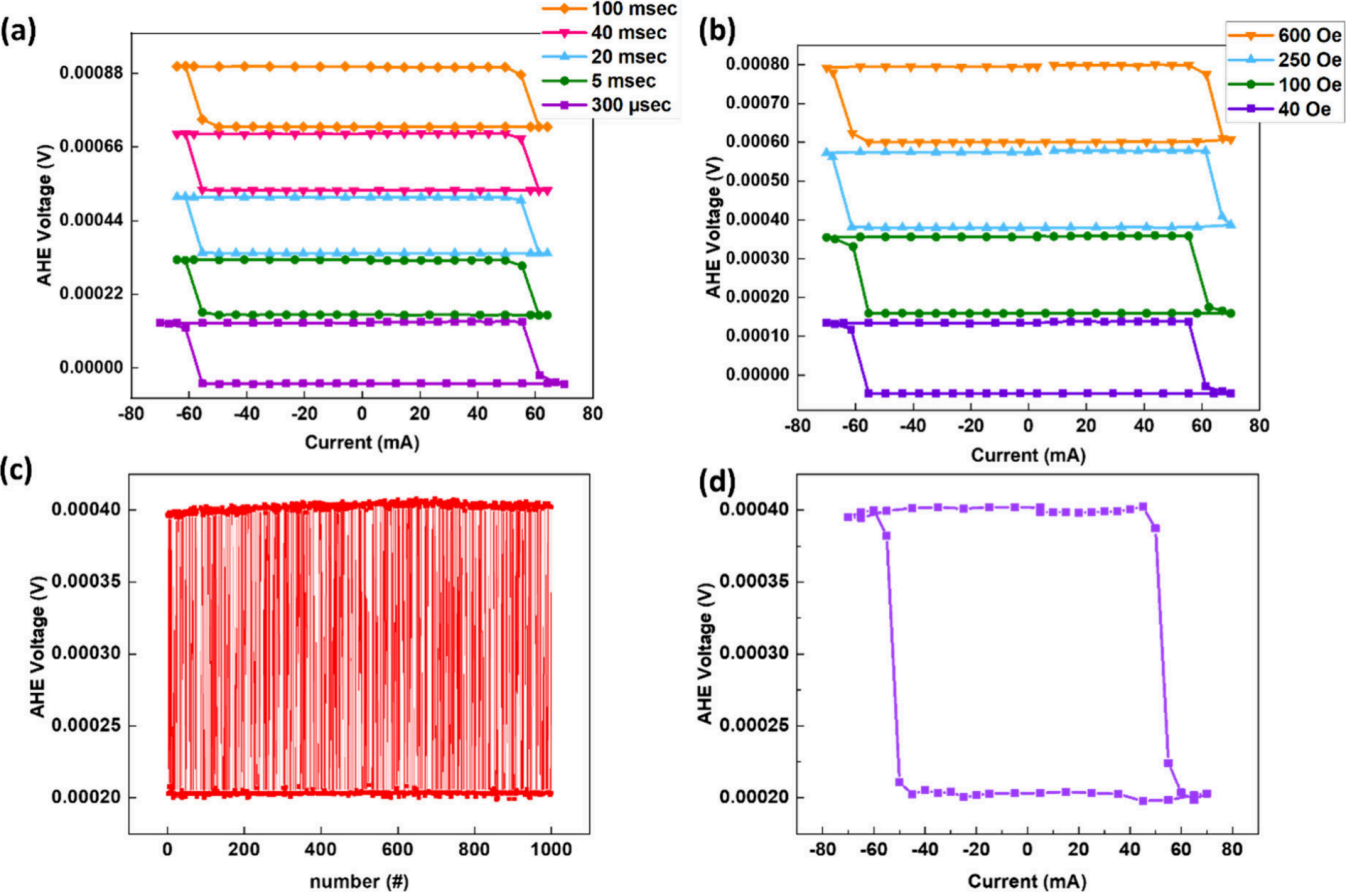
Stability of binary devices. (a) Effect of pulse width on the switching behavior under *H*_X_ = 40 Oe. (b) Effect of *H*_X_ on the switching behavior. Here each current pulse is 300 μs long. (c) Switching the binary device 1000 times by randomly applying 1000 consecutive current pulses of ±65 mA and 300 μs pulse widths under *H*_X_ = 40 Oe. (d) SOT switching loop after 3 (a), 3 (b), and 3 (c) switching tests; each pulse is 300 μs long, and this measurement is performed under *H*_X_ = 40 Oe.

Our devices exhibit dual SOT switching modes in the same device geometry irrespective of PtMn thickness, as shown in Supporting Information S5. We can still observe the dual SOT switching even when the PtMn was replaced by Pt, as shown in Supporting Information S6. All devices consistently exhibit binary switching initially and transition to multilevel mode only after applying current significantly larger than that required for binary switching. These results suggest that, unlike Fukami’s findings,^11^ dual SOT switching modes originate from microstructural changes in the (Co/Pd)_*n*_ multilayers and/or PtMn contingent upon current treatment, rather than exchange bias pinning effects by PtMn.

To clearly understand the transition mechanism, we performed high-resolution transmission electron microscopy (HRTEM) measurement to study the possible structural changes in our devices’ heterostructure after applying a large input current. Figure 3(a) and Figure 3(c) show the HRTEM analysis of the binary device (before the large current treatment). The HRTEM image of the heterostructure is shown in Figure 3(a), and Figure 3(b) shows the HRTEM image of the Co/Pd multilayer area. Based on the diffraction spots from fast Fourier transform (FFT) of Co/Pd lattice images of binary devices, as shown in the inset of Figure 3(b), we can verify that Co and Pd multilayers have formed an L_10_ ordered structure Co_50_Pd_50_ alloy with the tetragonal crystal lattice (*P*4/*mmm*). This is understandable, as the sample is annealed at 330 °C for 1.5 h, which promotes the formation of this L_10_ ordered Co_50_Pd_50_ alloy. Notice that no obvious twin structure was observed in the CoPd area of the binary device (before the large current treatment). Similarly, the HRTEM of the PtMn area, shown in Figure 3(c), and its diffraction spots from FFT, shown in the inset of Figure 3(c), reveal that PtMn is also the L_10_ ordered structure with the space group *p*4/*mmm*; no obvious twin structure was observed in the PtMn area either for the binary devices (before a large current treatment).

**Figure 3:**
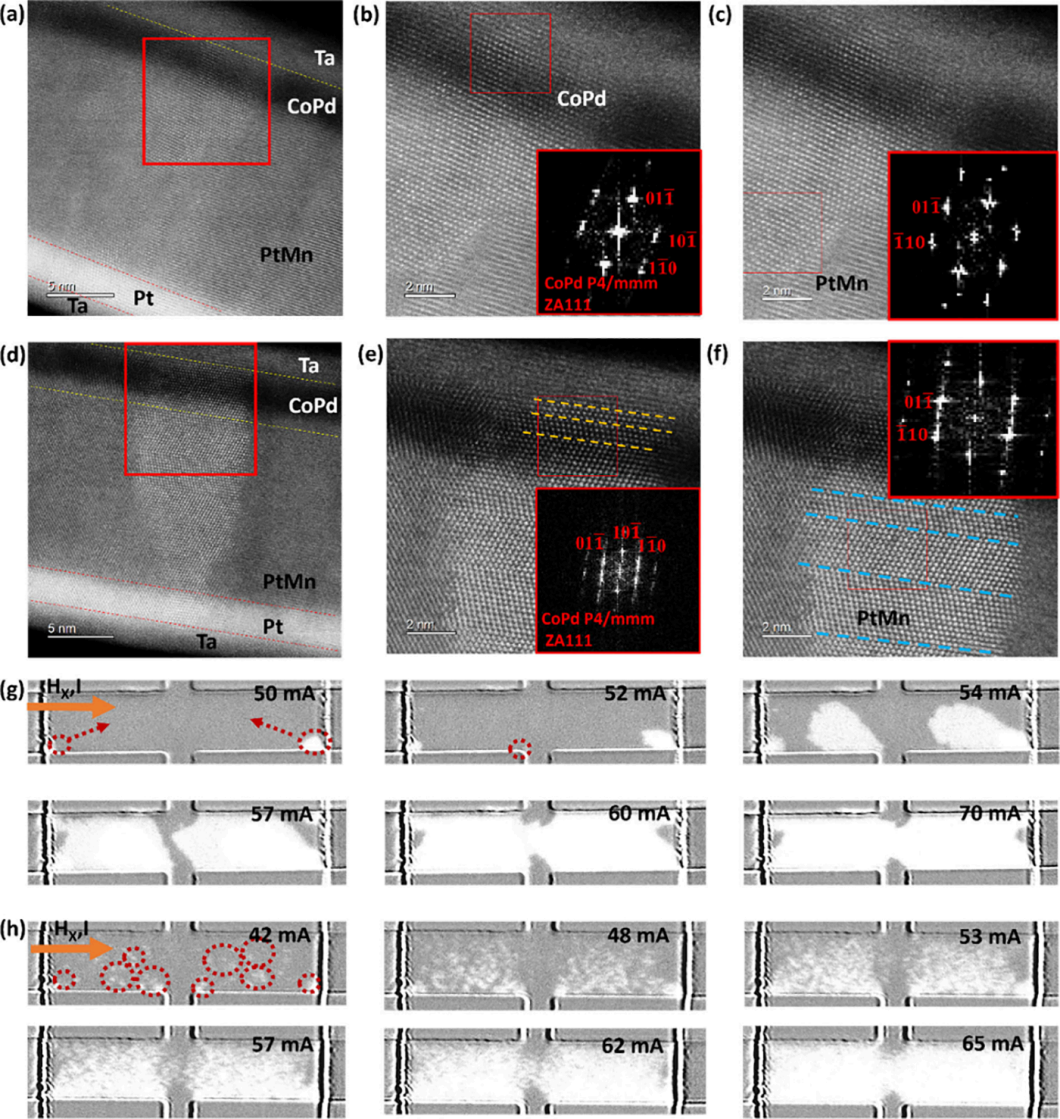
HRTEM analysis of binary and analog devices and switching dynamics for the two switching modes. (a) HRTEM image of the heterostructure of the binary device (before large current treatment). The red square indicates the analysis area for CoPd (b) and PtMn (c). (b) HRTEM image of Co/Pd multilayer area and its FFT (inset) to identify the diffraction pattern, which indicates the formation of ordered L_10_ Co_50_Pd_50_ (*P*4/*mmm*) alloys. (c) HRTEM image of PtMn area and its FFT (inset) to identify the diffraction pattern, which indicates the formation of ordered PtMn with a *p*4/*mmm* structure. (d) HRTEM image of the heterostructure of the analog device (after a large current treatment). The red square indicates the analysis area for CoPd (e) and PtMn (f). (e) HRTEM image of the Co/Pd multilayer area and its FFT (inset) to identify the diffraction pattern, which indicates the formation of ordered L_10_ Co_50_Pd_50_ (*P*4/*mmm*) alloys with a twin plane in (101), marked by orange dotted lines. FFT also shows the line-shape diffraction pattern. (f) HRTEM image of the PtMn area and its FFT (inset) to identify the diffraction pattern, which indicates the formation of ordered PtMn with a twin structure. Light blue dotted lines mark the twin planes. (g) Kerr imaging of the binary device depicting SOT switching by nucleation and propagation. (h) Kerr imaging of the analog device depicting SOT switching by nucleation. Each pulse is 300 μs long, and *H*_X_ = 40 Oe for in situ Kerr imaging.

Figure 3(d)–(f) show HRTEM analysis of the analog device (after a large current treatment). The HRTEM of the entire heterostructure is shown in Figure 3(d), and Figure 3(e) shows the HRTEM image of the Co/Pd multilayer area. Based on the diffraction spots from FFT of Co/Pd lattice images of the analog devices (after a large current treatment), as shown in the inset of Figure 3(e), we can verify that the L_10_ ordered Co_50_Pd_50_ alloy phase with the tetragonal crystal lattice was the same as that of the binary device. Notice that after the large current is input into the device, some of the CoPd area showed a twin structure with the (101) twin planes, marked by orange dotted lines in Figure 3(e). The twin structure was also revealed in the diffraction pattern (DP), in which the diffraction pattern became a line shape, as shown in the inset of Figure 3(e). Similarly, for the analog devices, we can also observe the formation of twins in the PtMn area, as shown in the HRTEM of PtMn (Figure 3(f)) and its deformed diffraction pattern shown in the inset of Figure 3(f). The twin structure of PtMn with twin planes is marked by the light blue dotted lines shown in Figure 3(f).

The in situ Kerr image for the SOT switching in the two modes is depicted in Figure 3(g) and Figure 3(h), respectively. It reveals that magnetization reversal changed from reversed domain nucleation and propagation (binary SOT) to nucleation-dominated (analog SOT) behavior. The twin structure formed in both L_10_ CoPd and L_10_ PtMn after a large current treatment can influence the magnetization reversal dynamics. More pinning sites for CoPd domain propagation may be induced by the formation of the twin structure of CoPd, or stronger pinning of CoPd domains may be caused by PtMn due to enhanced PtMn magnetic anisotropy, making the propagation of domain walls difficult. Therefore, magnetization reversal is dominated by domain nucleation without significant domain wall propagation, leading to analog switching after a large current treatment. For the binary devices (before a large current treatment), no obvious twin structure exists in CoPd or PtMn, resulting in magnetization switching through nucleation and propagation. Magnetization reversal by magnetic field and the corresponding in situ Kerr image for the two modes shown in Supporting Information S7 also show consistent switching dynamics like magnetization switching by current.

To further investigate the annealing effect on the switching mode, we also annealed samples at various temperatures up to 370 °C. As shown in Supporting Information S8, samples annealed up to 350 °C still revealed dual SOT modes, but samples annealed at 370 °C showed only the analog SOT after the annealing without any current treatment. While 370 °C thermal annealing can lead to the multilevel mode, all the devices on the wafer reveal only analog behavior. Using the current treatment method provides richer modulation and enables us to select devices to have binary or analog switching at the device level.

We used binary devices for artificial neuron behavior and analog devices for synaptic plasticity to demonstrate functionality. Figure 4(a) demonstrates the artificial neuron functionality in a binary device, leveraging thermal integration from successive current pulses. When pulses arrive closely, accumulated heat builds due to insufficient dissipation between pulses. This thermal buildup initiates magnetization changes through thermally assisted SOT switching, simulating biological neuron integration. Adjusting the pulse interval allows the device to mimic neuronal behavior as shorter time gaps between pulses increase the switching probability. To examine thermal integration effects, we choose pulses with pulse amplitudes of 56 mA and pulse widths of 10 μs, below the critical switching current of 60 mA for this pulse width; we varied time intervals from 300 μs to 10 μs between the successive pulses and found complete magnetization switching occurred with intervals under 30 μs and at least 20 pulses. This behavior aligns with sigmoidal neuron functions demonstrated in PtMn/(Co/Ni) nanometer size binary devices by Kurenkov et al.^10^ To demonstrate synaptic functionality, we used the analog device to show long-term potentiation (LTP) and long-term depression (LTD), as seen in Figure 4(b). Gradual changes in AHE voltage during Co/Pd multilayer switching represent the synaptic “weight”. Starting with magnetization at −*M*_Z_, we applied an external magnetic field (*H*_X_) of 40 Oe. For LTP, 27 consecutive negative pulses (300 μs each and a time gap of 1000 ms) were applied, with current gradually increased from 45 to 65 mA, resulting in a linear AHE voltage increase, mimicking synaptic strengthening. For LTD, 24 positive pulses decreased the AHE voltage linearly, simulating synaptic weakening. We repeated these measurements for multiple cycles. These AHE changes illustrate synaptic plasticity, which replicates essential neural functions. We have discussed the repeatability, robustness, linearity, and symmetry of LTP/LTD responses of synaptic weights in detail in Supporting Information 9.

**Figure 4:**
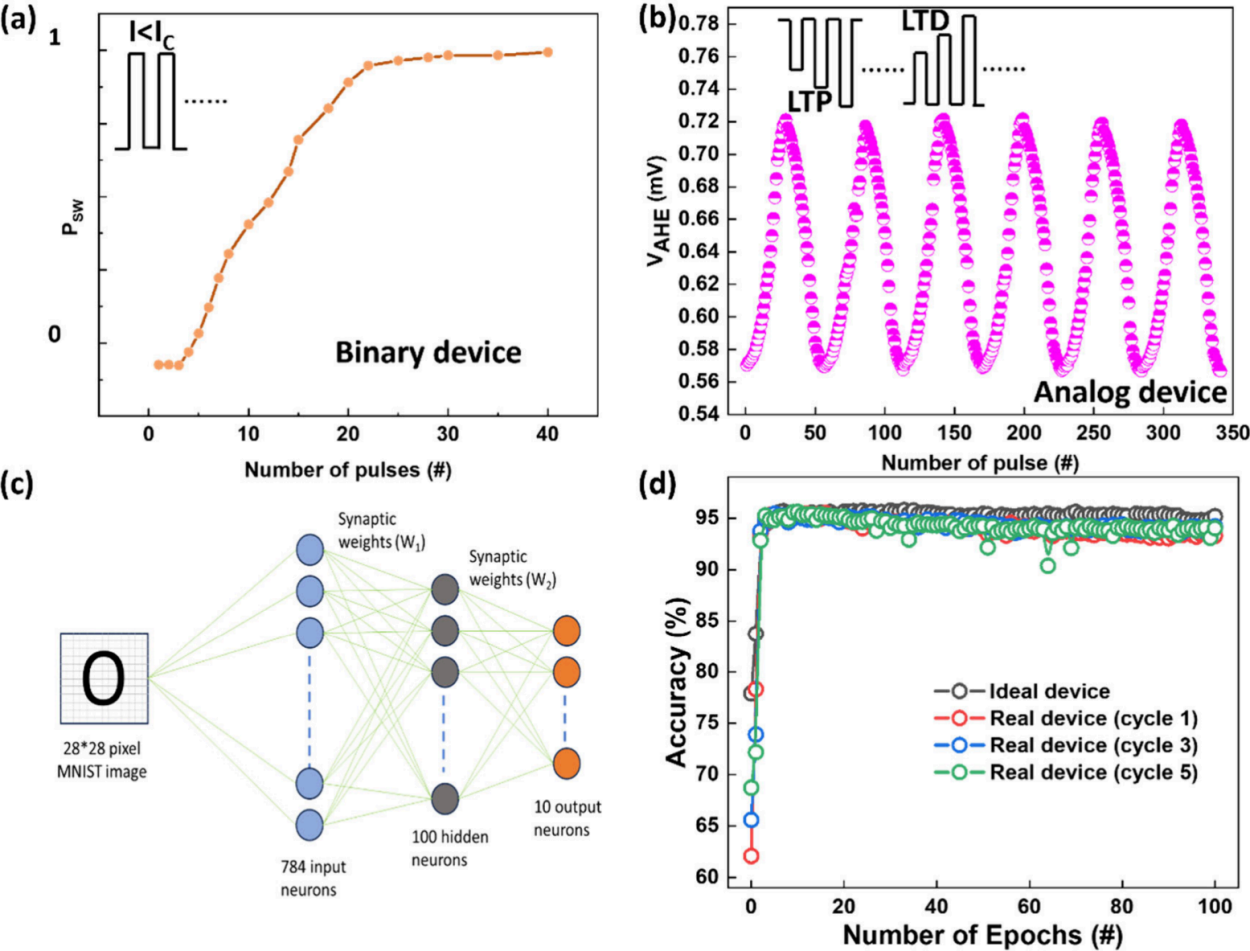
Demonstration of neuronal and synaptic functionalities in our devices. (a) Artificial neurons are demonstrated in binary devices by changing the number of current pulses applied. We used an input current of amplitude 56 mA, each pulse was 10 μs long with a time gap of 10 μs between the pulses, and the measurement was done under *H*_X_ = 40 Oe. (b) Demonstration of LTP and LTD in the analog devices achieved by increasing the number of pulses with increasing current amplitudes. The time gap between the pulses is 1000 ms, each pulse is 300 μs long, and the measurement was done under *H*_X_ = 40 Oe. (c) Multilayer perceptron (MLP) schematics used in the artificial neural network simulation for MNIST data set recognition. (d) Simulation results of Ta/Pt/PtMn/(Co/Pd)_4/_Ta with a recognition accuracy of ∼96% over three cycles.

To assess the neuromorphic potential of the PtMn/(Co/Pd)_4_/Ta device, we conducted ANN simulation to classify MNIST letters/digits using a multilayer perceptron (MLP) architecture.^39−42^ The MLP, shown in Figure 4(c), consists of three layers: an input layer with 784 neurons (one for each pixel in a 28 × 28 image), a hidden layer with 100 neurons, and an output layer with 10 neurons for digit classes 0 to 9. See Supporting Information S10 for a detailed explanation. The device’s synaptic behavior, characterized by its analog SOT curve/potentiation–depression curve (Figure 1(f) and Figure 4(b)), determines synaptic weights via corresponding AHE voltage states. Using these weights, the ANN was trained on 60,000 MNIST images and tested on 10,000 images over 100 epochs, achieving approximately 96% accuracy (Figure 4(d)) for three cycles. This accuracy aligns with recent works; 97–98.1% accuracy by Han et al. with CoPt/Ru/CoTb-based SAF,^20^ 91–96% by Ojha et al. with NiO AFM ordering,^21^ and 93% by Jiahao et al. with a compensated ferrimagnet.^26^

We have demonstrated a PtMn/(Co/Pd)_4_/Ta device capable of dual-mode SOT switching, binary and multilevel analog, achieved within a single, unmodified device stack structure and geometry. Binary switching is facilitated by nucleation and rapid propagation, while analog switching is realized through nucleation after high-current treatment. The transition from binary to multilevel SOT switching is attributed to structural changes in Co/Pd multilayers and the PtMn layer. These dual functionalities enable the device to act as both artificial neurons and synapses. Our ANN simulation achieved a remarkable 96% recognition accuracy on the MNIST data set. As dual-mode devices can be fabricated on a wafer, integration of neuron- and synapse-functioning devices is straightforward, leading to lower fabrication costs than prior works requiring distinct structures/process for each mode. This work demonstrates the potential for energy-efficient neuromorphic hardware, positioning this device as a promising candidate for advanced spintronic memory and computing applications.

We fabricated Si/SiO_2_//Ta(3)/Pt(2.5)/Pt_50_Mn_50_(20)/[Co(0.25)/Pd(0.66)]_4_/Ta(5) (thickness in nm) using magnetron sputtering, where Ta (3 nm) ensures adhesion and Pt (2.5 nm) enhances Pt_50_Mn_50_ (111) texture. Thicknesses were verified by atomic force microscopy (AFM). Samples with PtMn thicknesses of 4, 8, and 12 nm and reference samples (Si/SiO_2_//Ta(3)/Pt(5)/[Co(0.25)/Pd(0.66)]_4_/Ta(5)) without Pt_50_Mn_50_ but with 5 nm Pt were also fabricated. Films were deposited in Ar at room temperature (base pressure <2 × 10^–7^ Torr) and annealed at 330 °C for 1.5 h (<1 × 10^–5^ Torr). Magnetic properties were measured by using a vibrating sample magnetometer (VSM). Hall cross structures were patterned via photolithography and Ar ion milling, and devices were etched down to the bottom (SiO_2_). HRTEM structural analysis was performed using a spherical aperture-corrected field-emission transmission electron microscope (JEM ARM-200FTH).

## Data Availability

The data that support the findings of this study are available from the corresponding author upon reasonable request.

## Supporting Information Available

Magnetic properties characterized via VSM; quantitative estimation of DMI through *H*_X_-dependent SOT switching; stability and reproducibility of multilevel switching; influence of pulse width on SOT transition; PtMn thickness-dependent switching behavior; comparison of Pt vs PtMn underlayers; AHE and Kerr analyses of field-switched devices pre- and postcurrent treatment; impact of high-temperature annealing on dual-mode switching; repeatability, linearity, and symmetry of LTP/LTD; ANN modeling using AHE data as synaptic weights (DOCX)
