# Supplementary material for: Dual SOT Switching Modes in a Single Device Geometry for Neuromorphic Computing
Source: Nano Lett. 2025 Apr 17;25(17):7089–96. doi: 10.1021/acs.nanolett.5c01100 (PMC12046592; doi:10.1021/acs.nanolett.5c01100)
Supplement: Supplementary file 1 — nl5c01100_si_001.docx [file nl5c01100_si_001.docx]

**“Supporting Information for Dual SOT Switching Modes in a Single Device Geometry for Neuromorphic Computing”**

Abhijeet Ranjan^1^, Tamkeen Farooq^2^, Chong-Chi Chi^3^, Hsin-Ya Sung^5^, Rudis Ismael Salinas Padilla^1^, Po-Hung Lin^1^, Wen-Wei Wu^5,6^, Ming-Yen Lu^1,3,4^, Rahul Mishra^2^ and Chih-Huang Lai^1,4a*^

1. Department of Materials Science and Engineering, National Tsing Hua University, Hsinchu 30013, Taiwan.
2. Centre for Applied Research in Electronics, Indian Institute of Technology Delhi, New Delhi 110016, India.
3. Instrumentation Center, National Tsing Hua University, Hsinchu 300, Taiwan.
4. College of Semiconductor Research, National Tsing Hua University, Hsinchu 30013, Taiwan.
5. Department of Materials Science and Engineering, National Yang Ming Chiao Tung University, Hsinchu 300, Taiwan.
6. Future Semiconductor Technology Research Center, Hsinchu 30078, Taiwan.

^a*^Author to whom correspondence should be addressed: chlai@mx.nthu.edu.tw.

**Supplementary Information S1: Magnetic Properties.**

Figures S1(a) and S1(b) present the out-of-plane and in-plane hysteresis loops acquired via VSM. The loops exhibit robust perpendicular magnetic anisotropy, characterized by a squareness ratio of 1 and an anisotropy field (H_K_) exceeding 1 Tesla, which remains consistent before and after annealing. The strong perpendicular anisotropy has been observed in (Co/Pd) multilayers^1-3^. We did not observe an exchange bias since our annealing was performed without a magnetic field. Additionally, a slight increase in saturation magnetization was detected, possibly due to the interfacial ordering of the Co/Pd multilayers during annealing^3^. The coercivity increased from 250 Oe to approximately 400 Oe, indicating the formation of the L_10_ PtMn phase during the annealing process and the presence of exchange coupling between the (Co/Pd)_4_ and PtMn layers.


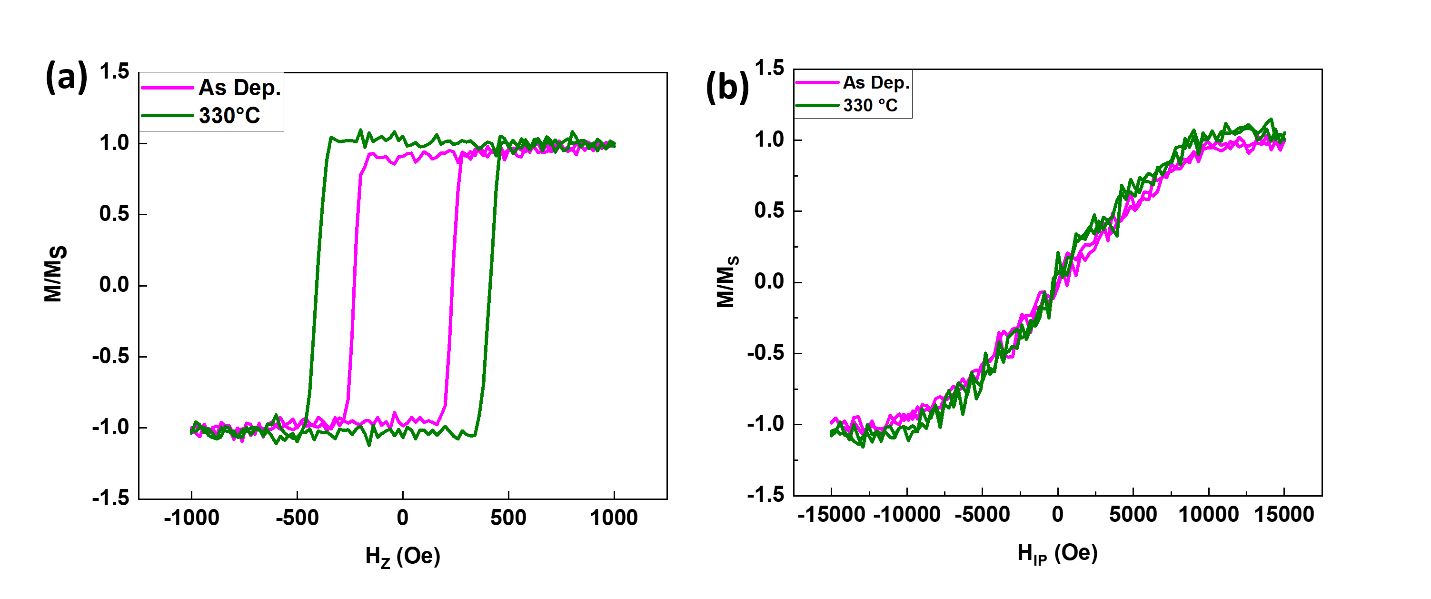


S1. Magnetic properties of the heterostructure: (a) Out-of-plane MH loop, (b) In-plane MH loop.

**Supplementary Information S2: Quantitative estimation of DMI.**

We performed H_X_ dependence of SOT switching study to quantitively estimate the HDMI for PtMn/Co/Pd and Pt/Co/Pd heterostructures. It is well known that to deterministically switch the magnetization using SOT, we must apply H_X_ sufficiently large to overcome the DMI barrier^4.^ This is shown below in Figure S2. Figure S2 (a) is for PtMn case, and Figure S2 (b) is for Pt underlayer case. As we can see in Figure S2 (a) for PtMn case, H_X_ between 25 Oe and 40 Oe are sufficient to cause SOT switching, while we can see in Figure S2 (b) that H_X_ required for fully switch the magnetization is between 240 Oe to 400 Oe. The pulse width for each pulse is 300 µsec. Thus, we can quantitatively estimate that DMI is much larger when underlayer is Pt as compared to when underlayer is PtMn. This measurement aligns well with reported values of DMI for PtMn/Co and Pt/Co interfaces +0.024 ± 0.005 mJ/m^2^ and −0.261 ± 0.008 mJ/m^2^ respectively^5^.

DMI can affect the SOT switching efficiency. The interfacial DMI stabilizes chiral Néel domain walls, which are more efficiently driven by spin-orbit torques. If the DMI is too weak, domain walls may become Bloch-type, less efficiently driven by spin-orbit torques. On the other hand, the **excessive DMI can pin domain walls or lead to the** Skyrmion **formation**, making it harder to drive switching with spin-orbit torque efficiently. Therefore, **moderate DMI is desirable to enhance SOT-driven switching efficiency** by stabilizing Néel walls and reducing critical current. The **Co/Pt interface** is well known for its **large interfacial DMI** due to the strong spin-orbit coupling of Pt and the broken inversion symmetry at the interface. **DMI strength in Co/PtMn** is **lower than in Co/Pt but still effective in driving the domain wall motion, as shown in Figure 3 (Kerr Image) for the binary devices. In addition, the reduced DMI also relieves the requirement of a large Hx, as shown in Figure 2.**


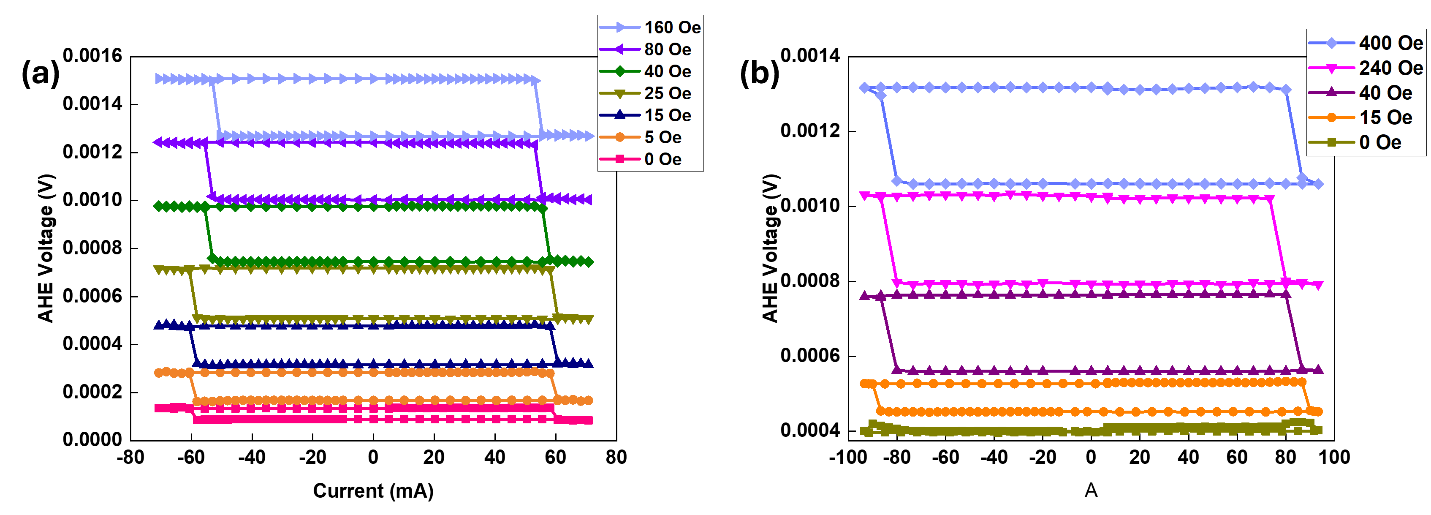


S2: H_X_ dependence of SOT switching for H_DMI_ estimation. (a) For PtMn case, H_X_ between 25 Oe and 40 Oe can achieve the full SOT switching, indicating presence of small DMI in PtMn/(Co/Pd) heterostructure (b) For Pt case, H_X_ between 240 Oe and 400 Oe can achieve the full SOT switching, indicating presence of small DMI in PtMn/(Co/Pd) heterostructure. Each current pulse is 300 µsec long for this measurement.

**Supplementary Information S3: Stability of multilevel.**

Once the irreversible transition to multilevel happened, the different intermediate states achieved were quite stable and reproducible, irrespective of how many times we did the same measurement. To clearly demonstrate reproducibility and stability of the intermediate state, the following measurements were carried out: we first saturated the magnetization at +M_Z_. After that, we randomly applied 140 current pulses with amplitudes between 46 mA and 64 mA; each current pulse was followed by a reset current pulse of -65 mA to make sure magnetization was at +M_Z_ state before the application of the next switching current pulse. Each current pulse was 300 µsec long, and the measurement was performed under H_X_ = 40 Oe. The measurement procedure is shown below in Figure S3 (a). As shown in Figure S3 (b), when we applied identical current pulses randomly, we reached the same intermediate states, confirming the reproducibility and stability of the intermediates of the analog devices.

**
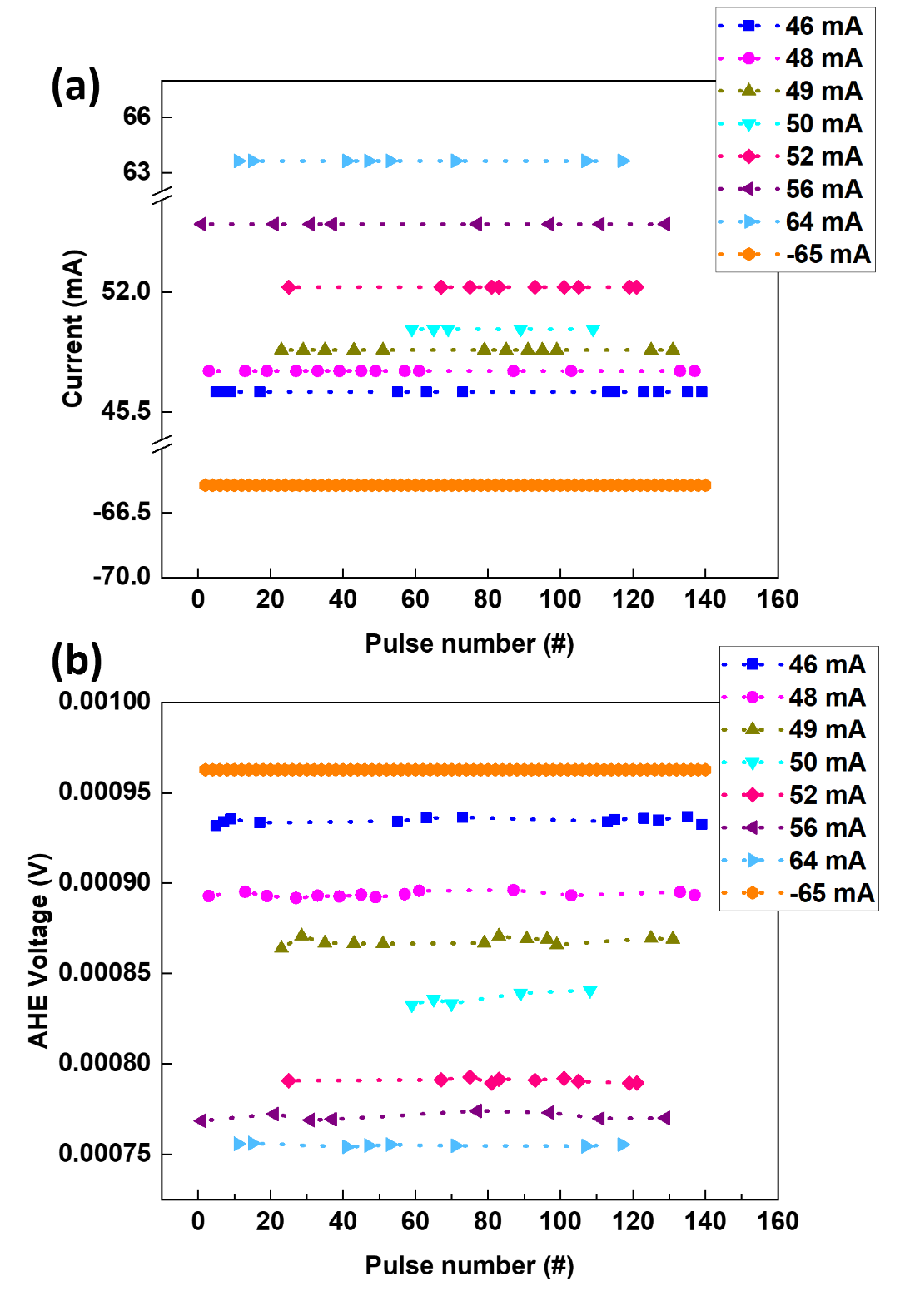
**

S3: Stability of intermediate states for the analog SOT devices. (a) Pulse sequence where we applied a pulse with a certain amplitude, which will cause the switching of the magnetization state; each pulse is followed by a reset current pulse of -65 mA to switch the magnetization back to +M_Z_, (b) reproducibility of intermediate states. Each pulse is 300 µsec long with an H_X_ of 40 Oe.

**Supplementary Information S4: Effect of shorter pulse width on transition.**

We performed SOT switching tests in the pulse width range of 10 µsec to 300 µsec. As shown in Figure S4 (a), the switching remained binary in this pulse width range as well. To further verify if we can get the transition by increasing the current amplitude at a shorter pulse width, we increased the current amplitude with a current pulse width of 30 µsec. As expected, once the current amplitude reached a certain threshold value, the transition indeed took place. Figure S4 (b) is SOT switching of the binary device using the current pulses width of 30 µsec. 70 mA was required to fully switch the magnetization in a binary manner. After this device was treated with a current of amplitude 88 mA and pulse width of 30 µsec, this device transitioned to the analog one. The analog SOT switching behavior under a current pulse width of 30 µsec is shown in Figure S4 (c). All the measurements were done under H_X_ = 40 Oe.


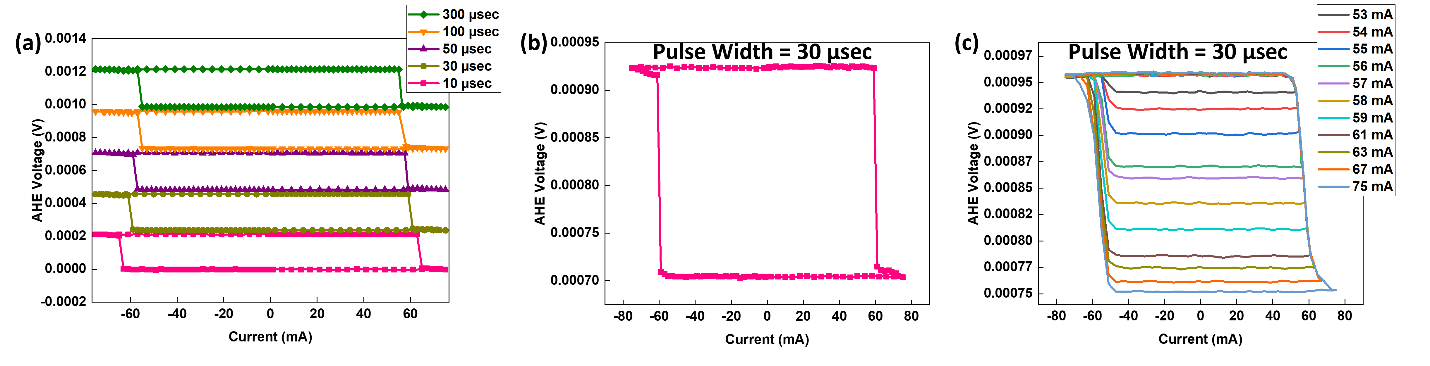


Figure S4: (a) SOT switching behavior in pulse width range of 10 µsec to 300 µsec. It remained binary in this pulse width range, (b) Binary SOT switching under a pulse width of 30 µsec; this device later transitioned to an analog one after it was treated with the current of amplitude 88 mA and pulse width of 30 µsec, and (c) Analog SOT switching under 30 µsec pulse width. All the measurements were done under H_X_ = 40 Oe.

**Supplementary Information S5: Effect of PtMn thickness on observed SOT transition.**

We tested the switching behaviors for thinner PtMn. We used samples with PtMn thicknesses 4 nm, 8 nm, and 12 nm. All samples were annealed at 330 °C for 1.5 hours. The SOT switching behaviors for these cases are shown in Figure S5. Like the PtMn 20 nm case, the same switching transition behavior (binary to multilevel) was obtained by treating the devices with a large current amplitude pulse in all three samples. Figure S5 (a), S5 (c) and S5 (e) correspond to the binary devices for PtMn thickness of 4 nm, 8 and 12 nm, respectively; Figure S5 (b), S5 (d) and S5 (f) correspond to multilevel devices for PtMn thickness of 4 nm, 8 and 12 nm after a large current treatment, respectively. The required current for the transition is 66, 69 and 80 mA for the PtMn thickness of 4 nm, 8 and 12 nm, respectively. All the measurements are done at H_X_ = 40 Oe with current pulse width of 300 µsec.

**
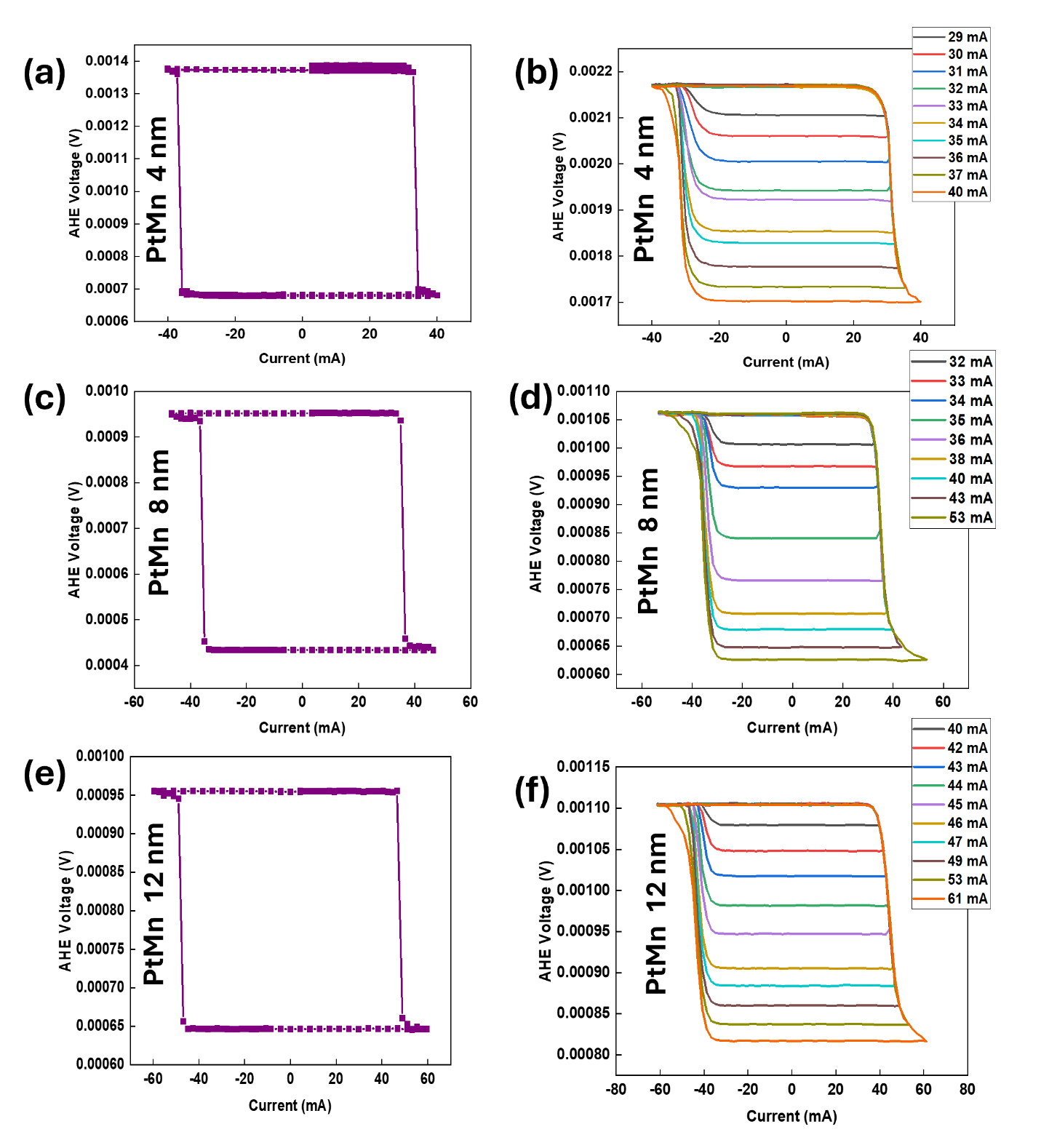
**

S5: SOT switching behaviors for different PtMn thicknesses before and after large current treatment. (a, b) Binary and analog SOT behavior in 4 nm thick PtMn, (c, d) Binary and analog SOT behavior in 8 nm thick PtMn, (e, f) Binary and analog SOT behavior in 12 nm thick PtMn. All the measurements are done at H_X_ = 40 Oe, and each current pulse is 300 µsec long.

**Supplementary Information S6: Replacing Pt with PtMn to observe SOT transition.**

To verify the role of PtMn in observed dual SOT modes, we replaced PtMn with Pt in our samples. In this Pt-based configuration, the devices exhibited even stronger perpendicular magnetic anisotropy (H_K_>1.5 T), as evidenced by the inability to saturate in-plane magnetization within our 15,000 Oe field limit. This result likely arises from the superior PMA at the Pt/Co interface compared to PtMn/Co. Figure S6 (a) confirms that the devices retain robust PMA after fabrication. As shown in Figure S6 (b), we achieved binary switching between up and down magnetization states with a 92 mA, 300 µs pulse under an external field H_X_ of 400 Oe, ten times higher than that required for PtMn-based devices, likely due to stronger DMI at the Pt/Co interface relative to PtMn/Co interface^5^. Increasing the current amplitude to ~113 mA resulted in resistance changes in the current channel, with SOT switching transitioning to an analog mode accompanied by increased device resistance R_XX_ (Figure S6 (c)). Figure S6 (d) illustrates the robust multilevel states achieved at different current amplitudes after the large-current treatment. Notably, after this transition, the H_X_ required to switch magnetization completely increased from 400 Oe to 1200 Oe, suggesting a possible change in DMI for the Pt underlayer. In contrast, in PtMn-based devices, the H_X_ needed for maximum switching remained stable at 40 Oe across switching modes. These results suggest that the dual-mode switching behavior in our devices can be attributed to the microstructural changes in (Co/Pd)_4_ multilayers during large current input to the device. However, parameters like DMI and the required external magnetic field H_X_ for SOT switching may vary depending on the underlayer material.


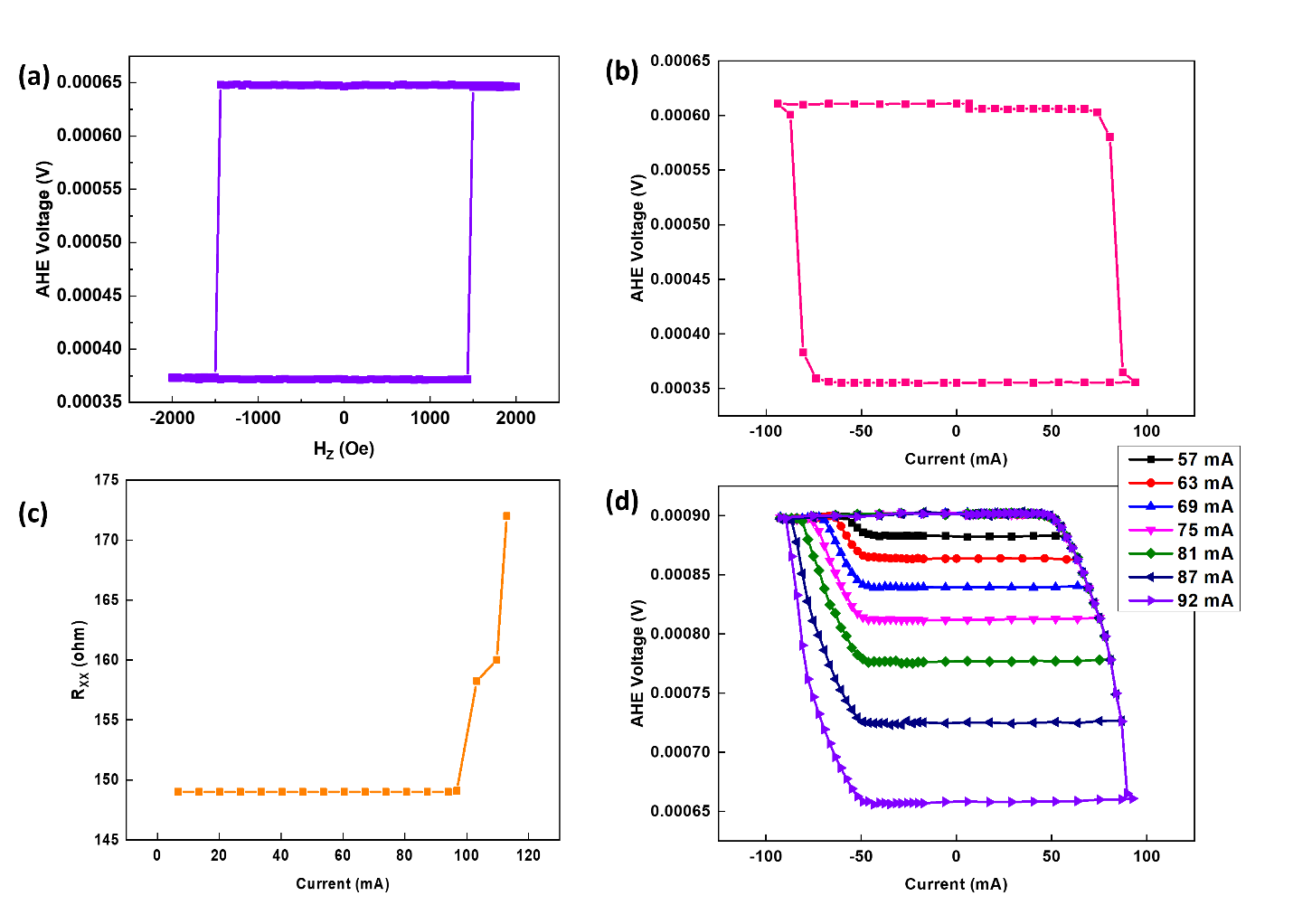


S6: SOT behavior in Pt/(Co/Pd)_4_/Ta. (a) AHE loop by field switching, (b) Binary SOT switching observed for maximum input current pulse of about 92 mA. Each pulse is 300 µsec long and H_X_ = 400 Oe, (c) Variations of resistance with the input current along the current channel (R_XX_), and (d) Analog SOT switching after a large current treatment of the device. Each pulse is 300 µsec long and H_X_ = 1200 Oe.

**Supplementary Information S7: Field switching for binary and multi-level devices.**

We compared the field-swept AHE loops before and after the high-current application to further investigate the mechanism behind the observed transition from binary to multi-level switching modes after a large current treatment. Supplementary Figure S7 (a) shows that the AHE loops indicate increased coercivity after high-current treatment, along with a sheared loop, instead of the sharp switching observed before the high-current treatment. This sheared loop shows that twin defects also make field switching more difficult as well possibly due to stronger pinning of CoPd domains after structural change. Figure S7 (b) shows the Kerr image of the device before the high-current treatment under an out-of-plane magnetic field of 600 Oe, while Figure S7 (c) displays the Kerr image for the device after the high-current treatment under the same conditions. Both devices reach full magnetization by switching with an applied field of 600 Oe. The device before the high-current treatment fully switches within approximately 4 seconds after field application, while the device after the high-current treatment requires around 9 seconds. This result suggests the domain walls are somehow pinned by the formation of twin defects during the high-current treatment. These pinning sites hinder both current-driven as well as field driven switching, which explains the transition from binary to multi-level SOT switching mode after large current treatment.


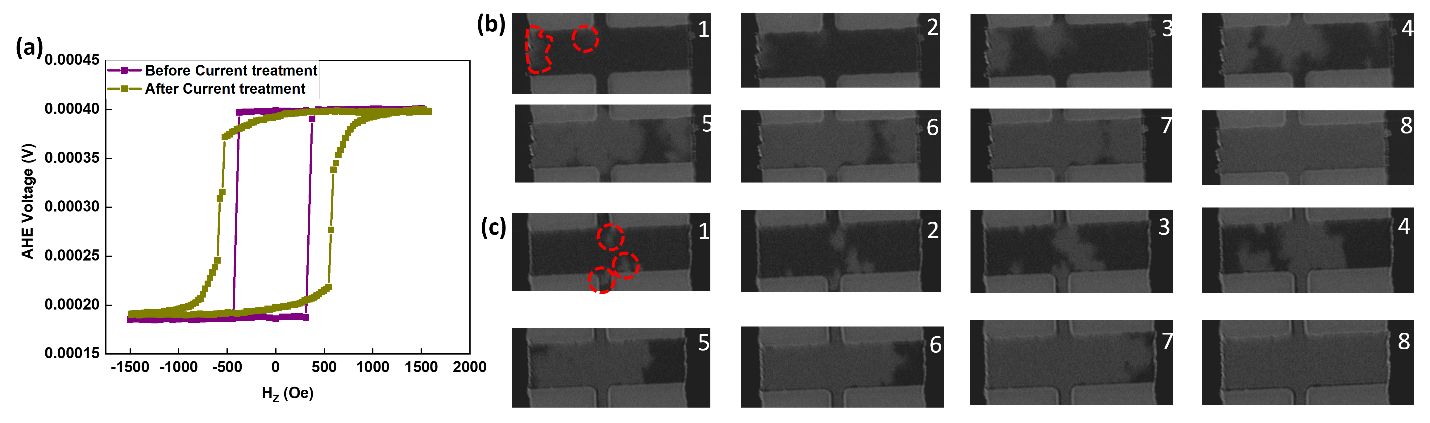


S7: AHE field switching for the device before and after a large current treatment, (b) Kerr image of the device before the high-current treatment under a 600 Oe out-of-plane magnetic field. (c) Kerr image of the device after the high-current treatment under the same field conditions.

**Supplementary Information S8: Effect of higher temperature annealing on SOT**

We tried annealing at various temperatures up to 370 °C for 1.5 hours and did the switching test; we observed identical SOT switching behaviors, i.e., the transition from binary to analog after a large current treatment for samples up to the temperature of 350 °C. Dual SOT modes obtained in the sample annealed at 350 °C are shown in Figures S8 (a) and S8 (b). The sample shows a binary SOT curve (Figure S8 (a)) with a current amplitude of 60 mA to fully switch the magnetization. The same device, when treated with a current amplitude of 75 mA, transitioned to an analog SOT device. The analog SOT curves for 350 °C annealed sample are shown in Figure S8 (b). For samples annealed at 370 °C for 1.5 hour, we observed the analog SOT switching after the annealing without any current treatment, as shown in Figure S8 (c). Figure S8 (d) shows the SOT switching dynamics for 370°C annealed sample using the in situ Kerr imaging during SOT switching. The switching takes place through the nucleation of the reversed domains. As discussed in Figure 3 of the main manuscript, the analog switching originates from the structural change of CoPd and/or PtMn due to the sufficient energy the large current treatment provides. Thus, a high thermal annealing temperature may provide a similar level of required energy for the structural changes to achieve the analog SOT switching.

Note that using the current treatment method provides richer modulation besides giving us the ability to select devices to have binary and analog devices at the device level instead of the wafer level, where all the devices will only have analog behavior.

**
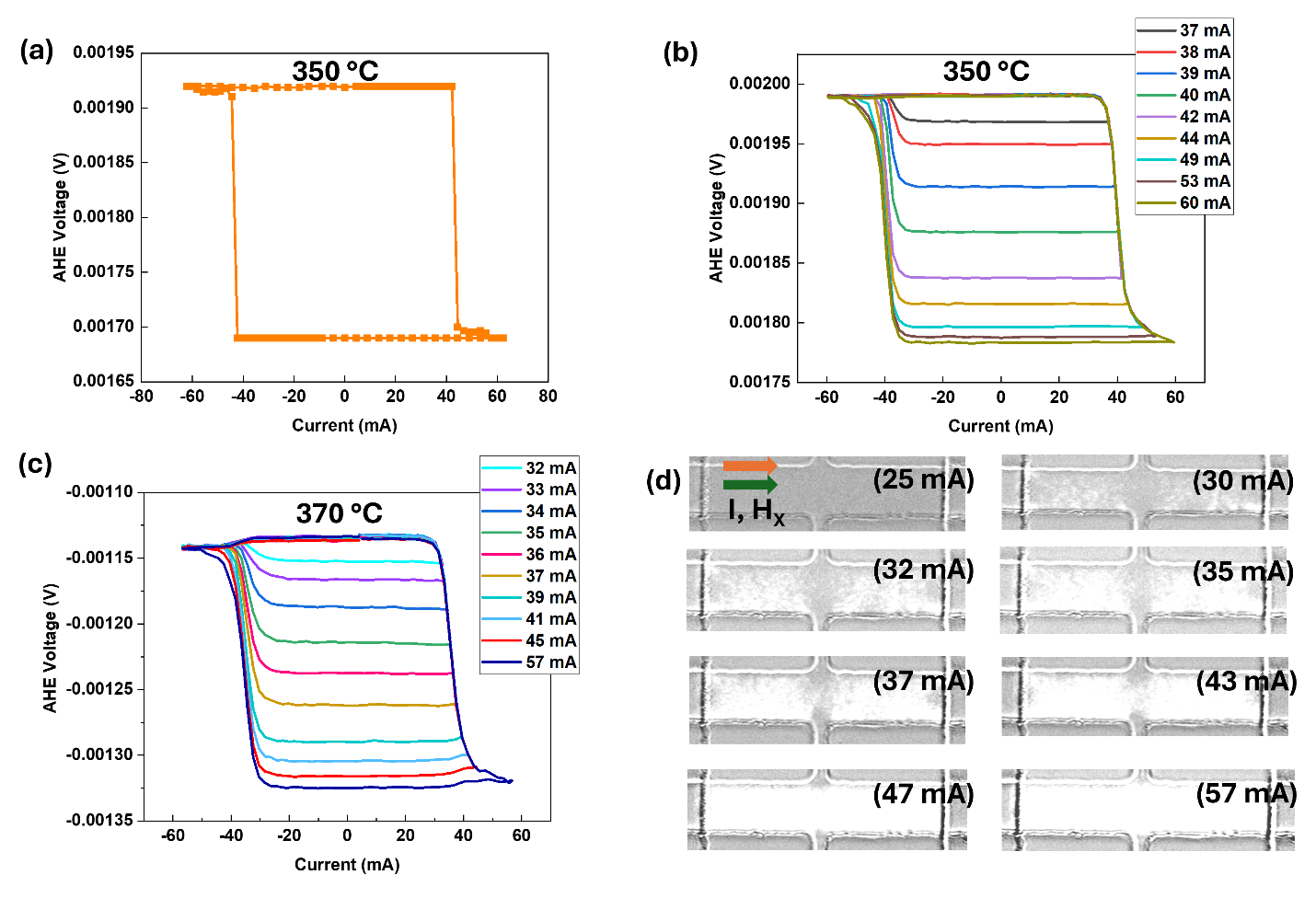
**

S8: Effect of higher temperature annealing on SOT. (a) Binary SOT switching behavior observed in the sample annealed at 350 °C (before large current treatment), (b) Analog SOT switching behavior observed in the sample annealed at 350 °C (after large current treatment (75 mA)), (c) Analog SOT switching behavior observed in sample annealed at 370 °C without any current treatment, and (d) Kerr Image of the analog SOT switching behavior in the sample annealed at 370 °C, Kerr images clearly show that this switching takes place through nucleation of the reversed domains. All the measurements are done at H_X_ = 40 Oe, and each current pulse is 300 µsec long.

**Supplementary Information S9: Repeatability, linearity, and symmetry of LTP/LTD.**

The repeated LTP/LTD measurements over multiple cycles are shown in Figure S9 (a). Starting from a magnetization state of −M_Z_, we applied an external magnetic field (H_X_) of 40 Oe. For the LTP process, 27 consecutive negative current pulses (300 μsec each with a 1000 msec interval), ranging from 45 to 65 mA, were applied. This resulted in a linear increase in anomalous Hall voltage (V_AHE_), simulating synaptic potentiation. Conversely, applying 24 positive pulses led to a linear decrease in V_AHE_, emulating synaptic depression. These measurements were repeated over multiple cycles, confirming the repeatability of the LTP/LTD behavior in our devices.

Next, we extract the variation of linearity and symmetry of LTP/LTD across multiple cycles of measurement. In Figure S9 (b), we plot the relationship between normalized V_AHE_ and normalized pulse number across various cycles of LTP/LTD, the data for which is extracted from Fig. S9 (a). From this data, the nonlinearity and symmetry variations of the potentiation and depression curves over different cycles are extracted and illustrated in Figures S9 (c) and S9 (d), respectively. The nonlinearity factor is calculated using the method given by Chen, Pai-Yu, et al^6^. The following equations are used to calculate the non-linearity factor (NL_LTP_ and NL_LTD_).

$G_{pot}=B\left( 1-e^{\left( -\frac{P}{A} \right)} \right)+G_{min}$

$G_{dep}=-B\left( 1-e^{\left( \frac{P-P_{max}}{A} \right)} \right)+G_{max}$

$B=-(G_{max}-G_{min})/\left( 1-e^{\left( \frac{-P_{max}}{A} \right)} \right)$

Here, $G_{pot}$ and $G_{dep}$ are the V_AHE_ values during potentiation and depression, respectively. P is the number of pulses, $P_{max}$ is the maximum pulse number, $G_{max}$ and $G_{min}$ represent the maximum conductance and the minimum conductance respectively Parameter *A* controls the nonlinear behavior of the synaptic weight update. *B* is a function of *A* that fits the functions within the range of $G_{max}$ , $G_{min}$ and $P_{max}$. Using a lookup table provided by Chen, Pai-Yu, et al^6^, the NL_LTP_ and NL_LTD_ are determined from the value of A. The calculated values of NL_LTP_ and NL_LTD_ are summarized in Figure S9 (c). The nonlinearity remains nearly constant across various cycles, indicating that the potentiation and depression characteristics of the device exhibit minimal fluctuation over repeated cycles.

The symmetry or the antisymmetric factor (AF) of the potentiation and depression curves is assessed by calculating the root mean square deviation (RMSD) between the LTP and LTD curves using the formulae:

$$AF=\sqrt{\frac{1}{N}\sum_{i=1} \left( f(x \right)-g(x)})^{2}$$

Here, *f(x)* and *g(x)* correspond to the potentiation and depression curve, respectively. The antisymmetric factor (AF) for various measurement cycles is plotted in Fig. S9 (d), which indicates that the symmetry of the LTP/LTD varies slightly across multiple measurement cycles. Overall, the antisymmetric factor of between the LTP and LTD is on the lower side. The higher AF values indicate greater asymmetry between the two curves.

These measurements reveal the repeatability of LTP/LTD characteristics of the device, especially for their linear and symmetric response. The digit recognition accuracy for different cycles is also analyzed. It is observed that there is very little variation in recognition accuracy when using different cycles of LTP/LTD weight update data, as shown in Figure 4d of the main manuscript. This aligns with the little variation in the AF and NL factors across multiple runs.

Figure S9: (a) Repeated LTP/LTD curve depicting the repeatability of the stability, linearity, and symmetry of synaptic weights update. (b) Normalized LTP and LTD curves over various cycles, (c) Non-linearity factor (NL) for the LTP/LTD for various measurement cycles, (d) Antisymmetric factor (AF) for various cycles of LTP/LTD measurements.

**Supplementary Information S10: Modeling AHE values as synaptic weights for ANN simulation.**

The neural network used is a three-layer artificial neural network (ANN) designed to map synaptic weights to experimental anomalous hall voltage (V_AHE_) data. The synaptic weights are normalized to a range of [-1, 1] to ensure compatibility with the V_AHE_ data range. This normalization facilitates the mapping of the weights to actual experimental V_AHE_ values through a linear transformation defined between the V_AHE-MIN_ and V_AHE-MAX_ values. Normalizing synaptic weights enhances learning efficiency, stability, and accuracy, leading to better overall performance in digit recognition tasks, as reported in the previous literatures^6-9^. To account for hardware imperfections, the V_AHE_ values are perturbed by introducing random noise and simulating stuck-at-minimum faults. These perturbations effectively model the variability in device behavior. Subsequently, the altered V_AHE_ values are mapped back to synaptic weights, enabling the ANN to adjust and adapt to these hardware-induced variations while preserving operational functionality.

**References:**

[1] Tudu, B.; Tian, K.; & Tiwari, A. Effect of Composition and Thickness on the Perpendicular Magnetic Anisotropy of (Co/Pd) Multilayers. Sensors 2017, 17(12), 2743.

[2] Kim, S.-K.; & Shin, S.-C. Alloy-like Co environment in Co/Pd multilayer films having perpendicular magnetic anisotropy. J. Appl. Phys. 2001, 89(5), 3055–3057.

[3] Gottwald, M.; Lee, K.; Kan, J. J.; Ocker, B.; Wrona, J.; Tibus, S.; Langer, J.; Kang, S. H.; & Fullerton, E. E. Ultra-thin Co/Pd multilayers with enhanced high-temperature annealing stability. Appl. Phys. Lett. 2013, 102(5), 052405.

[4] Wang, S. Y.; Chen, S. H.; Chang, H. K.; Li, Y. T.; Tseng, C. H.; Chen, P. C.; Yang, C.Y.; Lai, C. H. A Spin‐Orbit Torque Switch at Ferromagnet/Antiferromagnet Interface Toward Stochastic or Memristive Applications via Tailoring Antiferromagnetic Ordering. *Adv. Electron. Mater.* 2023, *9*(12), 2300472.

[5] DuttaGupta, S.; Kanemura, T.; Zhang, C.; Kurenkov, A.; Fukami, S.; Ohno, H. Spin-orbit torques and Dzyaloshinskii-Moriya interaction in PtMn/[Co/Ni] heterostructures. *Appl. Phys. Lett.* 2020, *111*(18).

[6] Chen, P. Y.; Peng, X.; Yu, S. NeuroSim: A circuit-level macro model for benchmarking neuro-inspired architectures in online learning. *IEEE Transactions on Computer-Aided Design of Integrated Circuits and Systems* 2018, 37, 3067–3080 .

[7] Ananthanarayanan, R.; Esser, S. K.; & Modha, D. S. 2014. *U.S. Patent No. 8,655,813*. Washington, DC: U.S. Patent and Trademark Office.

[8] Anwani, N.; & Rajendran, B. Normad-normalized approximate descent based supervised learning rule for spiking neurons. In *2015 international joint conference on neural networks (IJCNN)* 2015, July. (pp. 1-8). IEEE.

[9] Kim, S.; Lim, M.; Kim, Y.; Kim, H. D.; & Choi, S. Impact of synaptic device variations on pattern recognition accuracy in a hardware neural network. *Sci. Rep.* 2018, *8*(1), 2638.
